# 

**Published:** 1890-07-05

**Authors:** 


SATURDAY, JULY 5th, 1890.

**A Modern Miracle** . . . . . . . . . 193
**Words of Consolation.**—Hope . . . . . . 194
**The Doctor's Pulpit.**—Mountain or Sea? . . . . . 195
**The History and Capabilities of Herbal Simples.**—By W. T. Fernie, M.D. XIII.—The Cabbage . . . . 196
**Before the Lords' Committee.** VI.—Sir Sydney Waterlow (continued) . . . . . . . . . . 197
**Turning Over New Leaves** . . . . . . . 198
**Everybody's Page** . . . . . . . . . 199
**Notes and News** . . . . . . . . . 200
**Annotations.**—About Cucumbers—Normandy Butter—Tuberculous Cattle . . . . . . . . 202
**Can Tropical Africa be Colonised?**—By Sir Wm. Moore, K.C.I.E., Q.H.P. . . . . . . . . . 206
**The Practitioner's Mirror and Retrospect:**—
Medicine—A New Nasal Douche—Œdima Glottidis as a Result of Iodism—Insanity Cured by Influenza—Nitrate of Silver in Tabes, 203; The Bacillus of Diphtheria—Advantages of Warm Sublimate Solutions—Absorption of Fat . . . . . . . . . . . 204
Surgery—Injection of Varicose Veins—Resection of the Lungs . . . . . . . . . . . 204
Therapeutics—Resorcin—Sulphonal in Diabetes—Exalgin 205
New Drugs, Appliances, and Things Medical.—The "Rapid" Clinical Thermometer . . . . . 205
**July** . . . . . . . . . . . 207
**Passing Topics.**—"As Might be Reasonably Expected" . 208
**Scraps and Gleanings** . . . . . . . . 208

### EXTRA SUPPLEMENT—THE NURSING MIRROR.

**En Passant.**—Sydenham Nurses—Short Items—In Memory of the late Miss Fisher—Baby Farming—Halifax Fever Hospital—Sheffield Nurses' Home—Thrift in Philadelphia liii

**Notes from Australia** . . . . . . . . liv

**The Nurses' Bookshelf.**—The Training of Nurses in France . . . . . . . . . . . liv

**Everybody's Opinion.**—Lady Workers Wanted—A Home of Rest for Nurses—Fever Nurses—Infants and Castor Oil, lv.; A Seaside Holiday . . . . . . . lvi

**Presentations** . . . . . . . . . . lvi

**Vacancies—Notes and Queries** . . . . . . lvi

**Amusements and Relaxation** . . . . . . lvi

